# Tumor-Treating Fields Therapy for Pediatric Brain Tumors

**DOI:** 10.3390/neurolint13020015

**Published:** 2021-04-08

**Authors:** Atsushi Makimoto, Ryo Nishikawa, Keita Terashima, Jun Kurihara, Hiroyuki Fujisaki, Satoshi Ihara, Yoshihiko Morikawa, Yuki Yuza

**Affiliations:** 1 Department of Hematology/Oncology, Tokyo Metropolitan Children’s Medical Center, 2-8-29, Musashidai, Fuchu, Tokyo 183-8561, Japan; yuki_yuza@tmhp.jp; 2 Clinical Research Support Center, Tokyo Metropolitan Children’s Medical Center, 2-8-29, Musashidai, Fuchu, Tokyo 183-8561, Japan; yoshihiko_morikawa@tmhp.jp; 3 Department of Neuro-Oncology/Neurosurgery, Saitama Medical University International Medical Center, 1397-1, Yamane, Hidaka, Saitama 350-1298, Japan; rnishika@saitama-med.ac.jp; 4 Department of Neuro-Oncology, National Center for Child Health and Development, 2-10-1, Okura, Setagaya-ku, Tokyo 157-8535, Japan; terashima-k@ncchd.go.jp; 5 Department of Neurosurgery, Saitama Children’s Medical Center, 1-2, Shin-toshin, Chuo-ku, Saitama 330-8777, Japan; kurihara.jun@scmc.pref.saitama.jp; 6 Department of Pediatric Hematology/Oncology, Osaka City General Hospital, 2-13-22, Miyakojima-hondori, Miyakojima-ku, Osaka 534-0021, Japan; h-fujisaki@med.osakacity-hp.or.jp; 7 Department of Neurosurgery, Tokyo Metropolitan Children’s Medical Center, 2-8-29, Musashidai, Fuchu, Tokyo 183-8561, Japan; satoshi_ihara@tmhp.jp

**Keywords:** tumor-treating fields, TTFields, pediatric brain tumors, glioblastoma, clinical trial

## Abstract

Tumor-treating fields (TTFields) are alternating electric fields applied continuously to the brain by attaching two-pair arrays on the scalp. Although TTFields therapy has demonstrated efficacy against supratentorial glioblastoma (GBM) in adults, its safety and efficacy in children have not been confirmed. Despite differences in the genetic etiology of the adult and pediatric forms of GBM, both have certain clinical behaviors in common, allowing us to test TTFields therapy in pediatric GBM. Recently, several, pediatric case-series using TTFields therapy have been published, and a few, prospective, pediatric studies are ongoing. Because GBMs are extremely rare in pediatric patients, where they comprise a wide variety of genetic subtypes, these pediatric studies are feasibility studies targeting various types of malignant brain tumor. Although they are important for confirming the safety and feasibility of TTFields therapy in the pediatric population, confirming its efficacy against each type of pediatric brain tumor, including the GBM, is difficult. Our clinical research team, therefore, planned an investigator-initiated clinical trial targeting pediatric supratentorial GBMs (as in adults) with the aim of expanding regulatory approval of TTFields therapy for pediatric GBM treatment based on safety and exploratory efficacy data in combination with the accumulated evidence on adult GBMs.

## 1. Introduction

The glioblastoma (GBM), the highest grade of malignant astrocytoma, is one of the most aggressive types of malignant tumor as well as the most common primary central nervous system (CNS) tumor in adults. Its incidence is reportedly 3.23 per 100,000 in the United States (US) [1] and 4.64 per 100,000 in the United Kingdom [2] and increases with age. According to the Central Brain Tumor Registry of the United States (CBTRUS), GBM accounts for 14.5% of all primary CNS tumors (*n*= 415,411 in five years) while the proportion decreases to 2.9% in children and adolescents aged 0–19 years (*n*= 20,105 in five years) [1].

Treatment remains challenging, as GBM inevitably recurs despite the current, multimodal standard of care, including tumor resection followed by chemoradiation. Radiation therapy consists of 60 Gy in 30 fractions over a period of six weeks with concomitant daily temozolomide (TMZ) followed by adjuvant TMZ (days 1–5 every 28 days). The median survival time is reportedly eight months according to the CBTRUS registry and approximately 15 months in selected patients in clinical trials [3,4].

There are no comparable data on pediatric GBM because of its rarity. Although pediatric clinical trials are often conducted with eligibility criteria for both high-grade glioma (World Health Organization [WHO] classification grade 3) and GBM (grade 4), the prognosis of the patients is as poor as that of adults [5]. A standard treatment for pediatric GBM has not been established. Therefore, the same strategies used for adult GBM are usually applied to pediatric patients in clinical practice [5].

Tumor-treating fields (TTFields) therapy, a novel treatment modality utilizing alternating electric fields (AEF) with low-intensity and intermediate-frequency, has been approved by the Food and Drug Administration (FDA) in United States and its counterparts in other countries for treating GBMs. Because it works locally on the tumor site, the technique is similar to radiation therapy in part. However, AEF at low-intensity and intermediate-frequency differs from radiotherapy in being non-toxic to normal tissue. To optimize the quality of TTFields therapy, precise imaging studies are necessary to determine not only where the tumor is located but also how the AEF distributes from the insulated transducers placed on the skull surface. The present review article describes the introduction of this novel treatment modality and discusses its application to the treatment of pediatric brain tumors.

## 2. Mechanism of Action of TTFields

AEF, which are applied in various situations both in daily life and the clinical setting, show a wide range of effects on living tissue depending on their frequency. At very low frequencies under 1 kHz, which are usually delivered directly by contact electrodes, AEF can stimulate excitable tissues through membrane depolarization [6]. This phenomenon is used in low-frequency therapy, such as transcutaneous electrical nerve or muscle stimulation [6,7]. On the other hand, very high frequencies above 1 MHz causes tissue heating [8] and serves as the basis for several medical treatment modalities, including diathermy and radio frequency tumor ablation [9].

AEF with an intermediate frequency between kHz and MHz alternate too quickly to cause nerve-muscle stimulation or excessive tissue heating. Although they reportedly have no biological effects at low to moderate intensities [8], high intensities and intermediate frequencies can cause electropolation, which is applied in cell engineering in the laboratory [10]. In contrast, low-intensity AEF (<2 V/cm) and intermediate-frequencies (100–300 kHz) delivered via insulated electrodes showed a profound inhibitory effect on cell division in cultures as well as in murine tumor models.

This property of AEF was termed TTFields, and preclinical studies of its utility have been carried out by Novoure, Ltd. (Haifa, Israel). Kirson, et al., who took part in Novocure’s studies, identified two main mechanisms by which the electric fields may affect dividing cells [11].

First, the proper formation of the mitotic spindle is disrupted by moving tubulin dimers farther away from the growing end of the microtubules. This disrupts chromosome alignment and separation, resulting in mitotic arrest and cell death. Second, all the intracellular organelles and other particles are displaced to the mitotic cleavage furrow and accumulated there, thus interfering with cytokinesis and leading to the destruction of the cells exposed to TTFields. Furthermore, TTFields may also inhibit DNA damage repair, impair cellular migration and invasion [12], and upregulate autophagy [13]. The resulting daughter cells exhibit various forms of cell death including immunogenic cell death.

**Figure 1:**
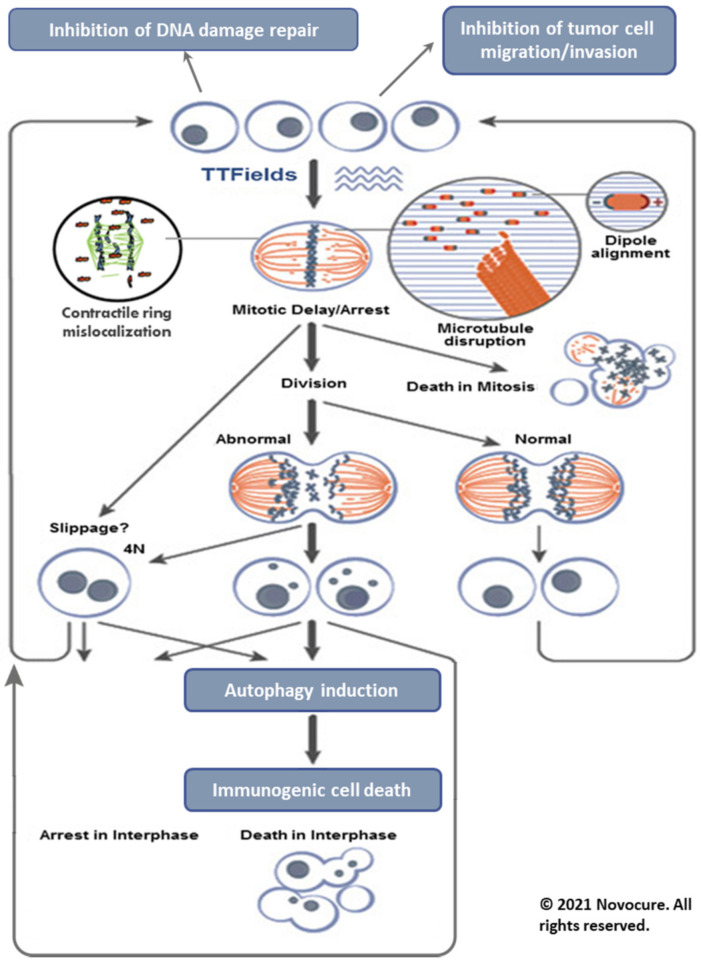
Mechanism of action of TTFields therapy. The TTFields exert directional forces on polar microtubules, interfere with the assembly of the normal mitotic spindle and subsequently trigger mitotic cell death. TTFields also inhibit DNA damage repair, impair cellular migration and upregulate autophagy, resulting in immunogenic cell death. Reproduced with permission from Novocure Inc [14]. Copyright 2021 Novocure—all rights reserved.

The mechanism of action is summarized and shown in Figure 1.

## 3. Preclinical Studies

Kirson et al. performed a series of preclinical studies prior to their clinical trial of TTFields. TTFields at 100 kHz (at an intensity of 1.0–1.4 V/cm) were found to have a profound inhibitory effect on the growth rate of a variety of human and rodent tumor cell lines (Patricia C, U-118, U-87, H-1299, MDA231, PC3, B16F1, F-98, C-6, RG2, and CT-26) whereas they had no inhibitory effect on non-replicating BHK cultures [11]. Another study demonstrated that the optimal frequency was 100 kHz for murine melanoma (B16F1), 150 kHz for human breast carcinoma (MDA-MB-231), and 200 kHz for glioma of rat (F-98) and human (U-118 and U-87) [15]. The effect on cell division and cell death by apoptosis is intensity-dependent, the sensitivity being highest for murine melanoma cells, lower for rat glioma and human non-small-cell lung carcinoma, and lowest for human breast carcinoma [15].

In vivo experiments with TTFields applied via electrodes implanted in intradermal tumors in C57BL/6 and BALB/c mice (B16F1 and CT-26 syngeneic tumor models, respectively) resulted in significant slowing of tumor growth and extensive destruction of tumor cells within three to six days [11]. In another study, the same team developed an animal model of intracranial glioma using Fischer rats which were intracranially inoculated with F-98 glioma cells using the stereotactic procedure [15]. The rats were treated with TTFields delivered by external electrodes at a frequency of 200 kHz at 2 V/cm for six days. Although the inhibitory effect was small with unidirectional (temporal-temporal) TTFields, increasing the number of TTField directions to 90 degrees showed significant inhibition of tumor growth [15].

The safety profile of TTFields in healthy animals was also investigated [15]. TTFields (100 kHz) at 6 V/cm were applied to the chest of three New Zealand rabbits. No changes were seen in the rate or regularity of the cardiac rhythm throughout and following exposure. To assess for chronic toxicity, TTFields were applied to either the head (*n* = 30, 1 V/cm for four weeks) or the chest (*n* = 10, 3 V/cm for two weeks) of New Zealand rabbits. All the animals were assessed weekly for weight, temperature, electrocardiogram, blood counts, and coagulation, and by wide chemistry panel. After a one-month follow-up period, all the animals were dispatched, and samples of the major organs were examined by a pathologist. No treatment-related toxicities were recorded in any of the animals [15].

## 4. Development of a Medical Device

The results of these studies prompted Novocure, Ltd. to manufacture Novo TTF-100A (product name: Optune^®^), a clinical TTFields device, for use in humans. The Optune^®^ system consists of two primary components, an electric field generator and two pairs of transducer arrays, each of which has a surface area of 22.5 cm^2^ and is placed on opposite sides of the head with the tumor positioned directly between them to deliver the TTFields non-invasively to the lesion. The portable field generator can be operated from an electrical outlet or by using a rechargeable battery.

The electrodes were completely insulated with ceramic material allowing the generation of adequate AEF without risking electrolysis on the electrode surface or intracellular ion concentration changes that accompany long-term current application [11,15]. The electrode configuration of Optune^®^ applies AEF at 200 kHz, 0.7 V/cm (Root Mean Square) to the center of the brain in two perpendicular directions for one second in each direction sequentially. The intensity was calculated using finite element mesh simulations and were verified by testing it on large animals and a human volunteer [11].

Precise placement of the transducer arrays is vital for Optune^®^ to exert optimal clinical efficacy. The current standard array placement configuration is designed to deliver TTFields to the supratentorial brain. The NovoTAL^TM^ software system developed by Novocure, Ltd. can draw the optimal array layouts based on head size, tumor location, and data from magnetic resonance imaging (MRI), including the characteristics of the tumor in each patient [16]. Preclinical studies have shown that the effects of TTFields increase with intensity, underscoring the critical need to understand how TTField-intensities distribute within a tumor.

**Figure 2:**
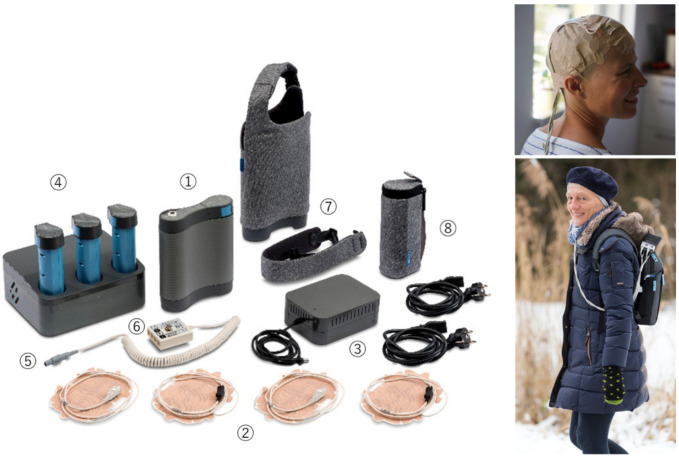
Optune^®^ Treatment Kit. The system contains an electric field generator to be connected to 2 pair of transducer arrays which are applied directly to the shaved scalp and a battery pack can be carried in a bag. 1. Electric Field Generator (the device), 2. Transducer Arrays, 3. Plug in Power Supply and Power Cords, 4. Portable Batteries and Charger, 5. Connection Cable, 6. Connection Box, 7. Shoulder Bag and Strap, 8. Reproduced with permission from Novocure Inc [14]. Copyright 2021 Novocure—all rights reserved. Permission for global image use was obtained from the patient.

The first-generation Optune^®^ device system included a field generator, transducers, batteries, and a carry bag weighing over 2.7 kg. A second-generation Optune ^®^ device, which weighs only 1.2 kg, was approved in 2016 [14]. This improvement enables patients to continue their normal daily activities while continuing TTFields therapy (Figure 2).

## 5. Early-Phase Feasibility Clinical Trials

A single arm, pilot trial of the safety and efficacy of TTFields therapy (EF-07) was performed in 20 patients with histologically proven GBM [15,17]. All the patients underwent multiple, four-week treatment courses using continuous-24-h, 200 kHz, 0.7 V/cm TTFields. The patients were divided into two groups according to their disease status.

The first group included ten patients with recurrent GBM [15]. All the patients underwent surgery and radiotherapy for the primary tumor. All but one patient received TMZ-containing chemotherapy as an adjuvant treatment prior to the recurrence. All the patients were treated with TTFields therapy for 280 weeks without any serious treatment-related adverse events. There were no significant changes in the serum chemistry or blood count in any of the patients. Only mild hepatic dysfunction, which was attributable to concomitant antiepileptic drug therapy, was observed. Two patients had partial seizures which were considered disease-related and unrelated to the TTFields therapy. Nine patients suffered from mild to moderate contact dermatitis caused by the transducer arrays, which was controllable with topical steroid and periodic electrode relocation.

The TTFields therapy resulted in a complete response in one patient, who is still tumor-free on MRI ten months after discontinuing the treatment, and one patient with a partial response, who maintained this response status for seven months after treatment discontinuation. Both patients have been progression-free for over two years from the start of the TTFields therapy. In addition, one patient had a minimal response, and four had stable disease for over four months before progression. The median progression-free survival (PFS) of the patients who received the TTFields therapy was significantly superior to that of the historical controls (26.1 weeks versus nine weeks, respectively). PFS at six months (PFS6) was 50% compared to 15% in the controls. As of the publication of the article cited [15], seven of the ten patients have died. The remaining three patients are alive, two of whom are progression-free. The median overall survival (OS) of the ten patients was 62 weeks.

The second group included ten patients with newly diagnosed GBM [17]. All the patients underwent surgery and radiotherapy for the primary tumor followed by TMZ as an adjuvant chemotherapy concomitantly with the TTFields therapy. The treatment was well-tolerated, and no serious treatment-related adverse events were observed. The patients averagely received the TTFields therapy for approximately 80% of the scheduled time. Mild to moderate contact dermatitis appeared at the sites of the transducer arrays in all the patients during treatment. In most cases, the dermatitis appeared early in the treatment course but responded well to the topical corticosteroid therapy. Periodic relocation to avoid skin irritation was also effective in increasing compliance with the TTFields therapy.

The median PFS of the ten patients was 155 weeks versus 31 weeks for the matched historical control patients who received maintenance TMZ alone. Two of the ten patients died. The remaining eight patients are alive, five of whom are progression-free as of the publication of the article cited [17]. The median OS from diagnosis was over 39 months versus about 14.7 months for controls treated with maintenance TMZ alone. Although the study was a small-size pilot trial enrolling only ten patients, excellent safety profile and highly promising efficacy data on TTFields therapy indicated the high therapeutic potential of TTFields therapy for newly diagnosed GBM.

## 6. Pivotal Clinical Trials

Two pivotal studies were conducted to evaluate the effectiveness and safety of TTFields, one in patients with recurrent GBM (EF-11) [18] and the other in patients with newly diagnosed GBM (EF-14) [4,19]. The results of these two pivotal studies are described below and summarized in Table 1.

EF-11 is a prospective, randomized, open-label, active controlled, parallel trial comparing the effectiveness and safety outcomes of TTFields alone in patients with recurrent GBM (*n* = 120) with the outcomes in patients receiving the best standard of care (BSC) with chemotherapy including CCNU, BCNU, and bevacizumab (*n* = 117), chosen by their respective physician [18]. The median OS, the primary endpoint of this trial, was 6.6 and 6.0 months (*p* = 0.27) for patients treated with TTFields and chemotherapy, respectively [18]. Although the trial did not demonstrate the superiority of TTFields therapy, it did demonstrate an efficacy at least comparable to that of commonly used chemotherapy regimens. However, TTFields therapy was superior in terms of the secondary endpoints; the response rate was 14% for TTFields vs. 9.6% for chemotherapy (*p* = 0.24), the median PFS was 2.2 for TTFields vs. 2.1 months for chemotherapy (*p* = 0.16), and the PFS6 was 21.4% for TTFields vs. 15.2% for chemotherapy (*p* = 0.13).

More importantly, the patients treated with TTFields alone experienced fewer adverse events in general, significantly fewer treatment-related adverse events, and significantly lower gastrointestinal, hematological, and infectious adverse events. The main, device-related adverse event was mild to moderate skin irritation at the transducer array sites, which was easily treatable with a topical medication. In addition, quality-of-life (QoL) metrics, including improved cognition and emotional well-being, were on the whole better in the patients with TTFields therapy than in the patients receiving BSC. The findings of the EF-11 trial led in 2011 to FDA approval of the first generation TTFields device for the treatment of GBM cases that are either recurrent or refractory to traditional therapy.

EF-14 is a randomized, open-label trial enrolling 695 patients with newly diagnosed GBM which was resected or biopsied. The patients completed concomitant chemoradiotherapy at one of 83 participating hospitals between July 2009 and July 2014 and were followed up through December 2016 [4,19]. Patients were randomized at a ratio of 2:1 to either a TTFields-plus-maintenance TMZ chemotherapy group (*n* = 466) or to a TMZ-only group (*n* = 229). TMZ was administered to both groups (150–200 mg/m^2^) for five days per 28-day cycle (6–12 cycles). Of the 695 patients (median age: 56 years; IQR: 48-63), 637 (92%) completed their treatment in the trial protocol. The primary endpoint was PFS, the secondary endpoints included overall survival and other efficacy and safety parameters, and intention-to-treat analysis was employed.

An interim analysis presented in 2015 demonstrated improved PFS (7.1 months in the TTFields-plus-TMZ group vs. 4.0 months in the TMZ-only group (*p* = 0.001)) [19]. Based on the data from the interim analysis, TTFields combined with TMZ was approved by the FDA in October 2015 for the treatment of newly diagnosed GBMs following maximal debulking surgery or biopsy and completion of concomitant radiation therapy. At the moment, there are several criticisms of the credibility of the EF-14 trials partly because the data reported were from the interim analysis at an intention-to-treat level. Therefore, the results of the final analysis are needed to determine the validity of the reported data.

After following up the study cohort until 2016, the results of the final analysis were published in 2017 [4]. The median PFS from randomization of the ITT population was 6.7 months in the TTFields-plus-TMZ group and 4.0 months in the TMZ-only group (hazard ratio (HR), 0.63; 95% CI, 0.52–0.76; *p* < 0.001), and the median OS was 20.9 months in the TTFields-plus-TMZ group vs. 16.0 months in the TMZ-only group (HR, 0.63; 95% CI, 0.53–0.76; *p* < 0.001).

The frequency of systemic adverse events was 48% in the TTFields-plus-TMZ group vs. 44% in the TMZ-only group. Mild to moderate dermatitis resulting from contact with the transducer arrays occurred in 52% of patients who received TTFields-plus-TMZ vs. none of the patients who received TMZ only. In the final analysis, the addition of TTFields to maintenance TMZ chemotherapy resulted in statistically significant improvement in progression-free survival and overall survival in line with the findings of the previous interim analysis.

There are inevitable lifestyle drawbacks because using the TTFields device requires shaving the scalp and wearing the arrays more than 18 h per day. The drawbacks and the treatment benefits have, of course, to be balanced. The secondary analysis of the EF-14 trial included health-related QoL questionnaires at baseline and every three months thereafter [20]. The health-related QoL scores did not differ significantly between treatment arms except for skin irritation. Deterioration-free survival was significantly longer in the TTFields-plus-TMZ group for the categories of global health (4.8 vs. 3.3 months, respectively; *p* < 0.01), physical functioning (5.1 vs. 3.7 months; *p* < 0.01), emotional functioning (5.3 vs. 3.9 months; *p* < 0.01), pain (5.6 vs. 3.6 months; *p* < 0.01), and leg weakness (5.6 vs. 3.9 months; *p* < 0.01), indicating that the addition of TTFields to standard treatment with TMZ resulted in improved survival without negatively influencing health-related QoL with the exception of more complaints of skin irritation attributable to the use of the transducers.

These findings led to the National Comprehensive Cancer Network (NCCN)’s adoption of TTFields therapy in the Clinical Practice Guidelines in Oncology for the Central Nervous System [21,22]. In 2018, the NCCN’s panel members designated TTFields therapy as a Category 1 treatment recommendation for patients with newly diagnosed GBM in conjunction with TMZ after maximal safe resection and completion of radiation therapy [21]. On the other hand, TTFields monotherapy for recurrent GBM was classified in 2015 as a Category 2B treatment, reflecting the absence of a consensus among the NCCN on the appropriate timing of the treatment [22].

## 7. Reliability and Feasibility of TTFields Therapy as a Standard of Care

After the FDA approved TTFields therapy for newly diagnosed GBM in 2015, the TTFields device has come to be used in many parts of the world including the EU and Asia. Nonetheless, skepticism about its reliability and feasibility persists [23,24]. Lassman et al. [24] recently reported the results of a survey of attendees at an educational session related to TTF during the 2019 meeting of the American Society of Clinical Oncology. Of the 30 respondents in total, 60% were convinced that TTF was able to prolong survival in patients with newly diagnosed GBM, but only 30% thought TTF should definitively be part of the standard of care treatment. Moreover, 87% opposed mandating TTF incorporation into the design of clinical trials [24].

Although reasons for this skepticism are multifactorial, the most important issue is the trial design of EF-11 and EF-14 and the interpretation of the results. EF-11, as discussed above, failed to demonstrate the superiority of TTFields therapy over chemotherapy. Although this is well-known among medical professionals, the Patient Registry Data set (PRiDe) [25], which will be discussed in the next chapter, may be seen as adding evidence corroborating the efficacy of the therapy in patients with recurrent GBM.

In terms of EF-14, there are mainly three issue to be discussed. First, the EF-14 had an open-label trial design without a “sham” device that might have led to false positive results due to the placebo effect. Although it is difficult to rebut this argument completely, the placebo effect is very unlikely to be seen in true endpoints like OS, which are key to demonstrating statistical significance (HR, 0.63 in the final analysis of EF-14) [4]. Moreover, clinical trials of a medical device using a placebo “sham” device for control patients would be very difficult to implement from an ethical perspective. The efficacy data should rather be complemented by subsequent, long-term observational studies.

The second criticism concerns patients’ selection. Wick [23] pointed out that the patients had already experienced a favorable course at the randomization because the median interval between diagnosis and randomization was 3.8 months. Because completion of the initial chemoradiotherapy without disease progression was the first criterion for trial participation, some patients who were not eligible for full-intensity chemoradiotherapy or who had rapidly progressive disease had to be excluded. Despite the known adverse risk factors, such as age, degree of surgical resection, and O^6^-methylguanine-DNA methyltransferase (MGMT) status, which factors might predict the subpopulation most likely to benefit from TTFields therapy is not known. In this regard, a well-designed clinical trial is warranted to identify patients who may benefit most from TTFields therapy and to determine the appropriate timing of the therapy.

Last but not least, the high cost of TTFields therapy is one of the biggest obstacles to its being included in the best standard of care. The total monthly cost of TTFields therapy for patients with GBM in the US is approximately 21,000 US dollars per month with regional variations and 1,500,000 Japanese yen per month in Japan. A French group concluded that the price of the TTFields device rendered the therapy far too expensive for routine use [26,27]. Hopefully, TTFields will become increasingly affordable as further development of the technology broadens its medical applications.

Due to these issues, it is currently difficult to include the TTFields therapy in the “standard of care” in routine medical practice. However, it may still be considered a viable treatment option for patients with GBM who do not have any contraindications for the therapy and are willing to try.

## 8. Studies of a Patient Registry and Post-Marketing Surveillance

The PRiDe, a registry of all patients with recurrent GBM who received TTFields therapy, was used to analyze the clinical outcomes of TTFields therapy at 91 cancer centers across the United States [25]. Compared to patients in the EF-11 trial, the 457 patients with recurrent GBM in the PRiDe data set were more likely to receive treatment with the TTFields device for their first recurrence (33% vs. 9%). The median OS resulting from the clinical use of the device showed improvement over the EF-11 trial result (9.6 months vs. 6.6 months; HR: 0.66; *p* = 0.003). A compliance rate for the use of the device of 75% or more versus < 75% was associated with significantly improved OS (7.7 vs. 4.5 months; *p* = 0.042). No significant adverse events were observed, with the most common side effect being skin irritation.

Recently, the results of a global post-marketing safety surveillance involving three regulatory bodies in the US, Europe (EU, Middle East, Africa), and Japan were published [28]. Unsolicited, post-marketing surveillance data from TTFields-treated patients from October 2011 to February 2019 were retrospectively analyzed, stratified by diagnosis (newly diagnosed GBM (ndGBM), recurrent GBM (rGBM), anaplastic astrocytoma/oligodendroglioma, other brain tumors) and age (<18 years [pediatric], 18–64 years [adults], ≥65 years [elderly]). Of 11,029 patients, 53% had the diagnosis of ndGBM, and 39% had the diagnosis of rGBM at any line of disease recurrence. Seventy-three percent were adults, 26% were elderly, and less than 1% (*n* = 52) were pediatric patients. The most commonly reported TTFields-related adverse event was array-associated skin irritation, which occurred in patients with ndGBM (38%), rGBM (29%), anaplastic astrocytoma/oligodendroglioma (38%), and other brain tumors (31%) as well as in 37% of pediatric, 34% of adult, and 36% of elderly patients. Most skin adverse events were mild to moderate and manageable. Other TTFields-related adverse events in patients with ndGBM or rGBM included discomfort caused by heat generated by the arrays (warmth: 11%, 10%, respectively), a tingling sensation related to the electric currents (tingling: 11%, 9%, respectively), and headache (7%, 6%, respectively). The TTFields safety surveillance analysis, which included more than 11,000 patients, revealed no new safety concerns and showed a favorable safety profile comparable with that of previous TTFields/GBM trials.

The safety profile of TTFields therapy in these studies, confirmation of the efficacy of the therapy against newly diagnosed or first-recurrence GBM, and the real-world experience of pediatric patients demonstrating an equivalent safety profile as in adults justify the use of TTFields therapy in pediatric patients with GBM in a clinical trial setting.

## 9. Development of TTFields Therapy for Pediatric GBM

### 9.1. Characteristics of Pediatric GBM

The similarities and differences between pediatric and adult GBM are very controversial, especially with regard to the divergences seen in the genetic profiling of individual patients. The WHO pathology classification of 2007 treats both types of GBM as a primary (or de novo) GBM, that is, as a single disease entity [29]. Pathologically, GBM is a malignant tumor of glial cell origin featuring nuclear atypia, cellular pleomorphism, mitotic activity, a typically diffuse growth pattern, and microvascular proliferation and/or necrosis [29,30]. Both the adult and pediatric forms have the same morphological findings. Therefore, pediatric neuro-oncologists use the same treatment strategies as those used for adult patients with GBM.

In addition to the morphological features, the 2016 WHO classification uses a wide variety of genomic abnormalities, which were discovered in both pediatric and adult GBM, to characterize the disease more accurately [30]. One of the most important driver mutations in GBM is *IDH*, which is usually a wild-type gene in pediatric GBM. Besides *IDH*, there are various, other mutations that can be divided into three groups. The first group consists of *EGFR*, *TERT*, and *PTEN*, which are frequently detected in adult GBM. The second group consists of *NTRK*, *H3K27M*, *H3G34R*, and *H3G34V*, which are frequently detected in pediatric GBM. Finally, there is a mutation group common to both adult and pediatric patients with GBM, including *RAS*, *MAPK*, *RB*, *P53*, and genes related to various tyrosine kinase receptors. The accumulating evidence may contribute to devising effective treatment strategies using so-called precision medicine. Thus far, however, the treatment modalities available for treating pediatric patients with GBM are limited.

The US clinical trial ACNS0126, which evaluated the efficacy of the standard, multidisciplinary treatment comprising surgical resection and chemoradiotherapy with TMZ followed by TMZ maintenance therapy, resulted in a three-year survival rate of 22% and a three-year PFS of 11% in all the subjects, including those with GBM and high-grade glioma (HGG) [5]. Although these data were moderately better than those for adult GBM, the three-year PFS of 55 pediatric patients with GBM was as low as 7% (there is no information on the three-year survival rate). Also, data from several patient registries showed evidence of poorer prognosis of GBM in pediatric patients than in adult patients. In the British National Cancer Registry Service, the median survival time was 12, 26, and 17 months in patients aged 0–19 years, 20–29 years, and 30–39 years, respectively [2]. The US data in the CBTRUS showed similar outcomes. The five-year survival rate was 19.6%, 26.8%, and 5.9% in patients aged 0–14 years, 15–39 years, and 40 years or older, respectively [1].

### 9.2. Rationale for Expanding the Use of TTFields Therapy for Pediatric GBM

Because of the rarity and severity of pediatric GBM [1,2,5], development of a novel treatment is extremely challenging but absolutely necessary. Although there is a trend in the age distribution of the tumor genotypes in GBM, the phenotype, including microscopic morphology and clinical tumor behavior, are common to adult and pediatric GBM. Because it is standard practice to apply the treatment strategies for adult GBM to pediatric cases, the application of TTFields therapy to pediatric GBM is considered reasonable. The effectiveness of TTFields therapy is a result of an interaction between its physiological activity and tumor cell division. Therefore, differences in the tumor genotype should not be an important factor influencing the effectiveness of this therapy.

Branter et al. presented an assessment of the efficacy of TTFields in pediatric brain tumor cell lines including GBM, medulloblastoma, and ependymoma at the 2018 Society for Neuro-Oncology conference [31]. A panel of tumor cell lines were treated with a range of clinically relevant frequencies (100–400 kHz) for 72 h to determine the optimal frequency for each cell line. TTFields showed significant efficacy against all the cell lines, with the degree of effectiveness being dependent upon the frequency used. The treated cells demonstrated up to 75% slower growth rates following treatment. Cell cycle analysis revealed that cells treated with TTFields had significantly greater levels of G2/M phase accumulation relative to the controls in line with previous findings in adult GBM cell lines. The authors concluded that TTFields demonstrated efficacy against pediatric brain tumors, including pediatric GBM, and that further investigation was warranted to develop a clinical application.

According to the global post-marketing safety surveillance described above, in total 52 pediatric patients (age < 18 years) were treated with TTFields therapy [28]. The incidence of skin irritation caused by the transducer arrays was similar (approximately 35%) among pediatric, adult, and elderly patients. The incidence of all adverse events was lowest in the pediatric patients, most likely due to the smaller sample size. No serious adverse events were reported in the pediatric patients.

Currently very few clinical studies of pediatric cases are available. A comprehensive search of articles in PubMed found two case reports [32,33] and one abstract presented at an academic conference [34]. Table 2 summarizes the clinical characteristics of all the reported cases. The background of the patients was so diverse as to render objective assessment of treatment efficacy impossible. However, the safety profile of pediatric patients was favorable, showed no unexpected toxicities, and was comparable to that of adult patients.

Taken together, clinical trials of TTFields for pediatric brain tumors is warranted. Because the most important issue in pediatric clinical trials is ensuring the safety of the patients, researchers need to monitor the condition of their subjects to guard against possible, unexpected toxicities.

### 9.3. Ongoing Clinical Trials to Test Optune^®^ for Pediatric Brain Tumors

The database of the National Cancer Institute trial registration (clinicaltrials.gov: accessed on 25 January 2021), contains only two active clinical trials of Optune^®^ for pediatric brain tumors.

One is a multi-institutional feasibility trial conducted by the Pediatric Brain Tumor Consortium (PBTC-048) [35] with the primary objective of examining the feasibility and device-related toxicities of Optune^®^ in children aged 5–21 years with supratentorial HGG (including GBM) or ependymoma. The secondary objectives included the response rate, event-free survival, compliance, and QoL. According to the abstract presented at the 2018 Society for Neuro-Oncology conference [36], the planned interim analysis included 11 patients with supratentorial tumors, of whom ten had HGG and one had ependymoma. The median age was 14.2 (6.4–21.3) years. Except for one patient in whom progressive disease occurred in the very early phase of the treatment, ten patients were evaluable, and four remained in the study through four treatment cycles, with one patient on cycle 14 at the time of the conference. One grade 5 intracranial hemorrhage was not associated with use of the device and no grade IV toxicities were found, and three patients experienced seizures (grade 1–3), fatigue, scalp pain, localized rash, or headache (none greater than grade 3). Of the ten evaluable patients, seven satisfied the feasibility criteria for Optune^®^ therapy, which is above the prespecified threshold of at least six of 11 patients. The preliminary results indicated feasibility of the treatment and minimal toxicity. The study is ongoing and aims to recruit 25 patients in total.

The other clinical trial was a small feasibility trial (*n* = 6) of Optune^®^ with TMZ and bevacizumab for pediatric HGG and ependymoma conducted at two centers [37] with the primary objective of evaluating the safety and tolerability of treatment using Optune^®^. The secondary objectives were the PFS and the OS of patients to aid in the future development of large-scale studies using Optune^®^ treatment. Although this trial reportedly began in April 2017, none of the results has yet been published.

### 9.4. Objectives and Goals of our Pediatric Clinical Trial

Our team plans to launch an investigator-initiated clinical trial named “A safety/efficacy trial of NovoTTF-100A (Optune^®^) for pediatric glioblastoma” in March 2021. In Japan, off-label use of medical devices is almost impossible because the national health insurance system does not cover the cost of off-label use of drugs and medical devices and associated supportive care. Therefore, pediatric neuro-oncologists and neurosurgeons in Japan cannot apply TTFields therapy to the treatment of pediatric GBM even if the scientific evidence is sufficient to warrant its use.

Due to such regulatory issues in Japan, the target population of the scheduled trial is limited to pediatric patients with GBM. The goal of the trial is to overcome the regulatory hurdles for expanding the use of the second-generation Optune^®^ for pediatric GBM treatment, that is, to effect at least a partial change in the regulatory status and labeling of Optune^®^ for the treatment of pediatric GBM. Given the rarity of pediatric GBM (less than 30 patients per year in Japan), only a small trial is likely to be feasible. Discussions with the Pharmaceuticals and Medical Devices Agency (PMDA), the regulatory agency in Japan, led to a tentative consensus that the accumulated data on the efficacy of Optune^®^ for adult GBM may be extrapolatable to pediatric GBM if the trial is able to demonstrate efficacy equivalent to that found in previous, adult studies. On the other hand, the combination of the pediatric safety data gathered in this trial and the findings of international studies, including clinical trials and post-marketing surveillance studies, may expedite approval of the device for pediatric GBM treatment.

The scheduled trial aims to evaluate the safety and efficacy of NovoTTF-100A in pediatric patients with GBM. The inclusion criteria are (1) histopathological diagnosis of GBM; (2) the main disease site in the supratentorial region; (3) age between 5 and 17 years; (4) newly diagnosed or first-recurrence GBM; (5) completion of clinically indicated surgical resection of the tumor; (6) completion of clinically indicated radiation therapy or completion expected within 14 days from enrollment, (7) Karnofsky/Lansky performance status equal to or more than 70; and (8) written informed consent from the patient and/or legal guardian.

All the patients will receive TTFields therapy using NovoTTF-100A for 28 days per course for up to 26 courses until the end-of-therapy criteria are met. Concomitant treatment with chemotherapy will be allowed. The primary endpoint is the adverse event rate with causality. The secondary endpoints include the response rate, clinical benefit rate, PFS rate (six months and one year), OS rate (one and two years), PFS, OS, QoL, and adverse event rate. In view of the unavoidable lifestyle drawbacks of wearing the transducer arrays all day, QoL will be evaluated in detail using PedsQL^TM^, including both the Core scale and the Brain Tumor Module.

In total ten patients will be enrolled from March 2021 through March 2024. Follow-up observation will end in September 2025. Registration with the Japan Registry of Clinical Trials (jRCT) is currently pending confirmation by the Ministry of Health, Labour and Welfare of Japan so that the study treatment can be overseen by the Advanced Medical Care administration system prior to official announcement of trial commencement.

### 9.5. Future Promise of TTFields Therapy in Pediatric Oncology

The future development of TTFields therapy is expected to be bidirectional and include an expansion of applicable anatomical sites as well as of types of treatable neoplasm. In terms of the former direction, the standard array placement configuration is designed to deliver TTFields to the supratentorial area. For pediatric neuro-oncologists, the potential of TTFields therapy for posterior fossa tumors is of greatest interest because of the high incidence of these tumors, including medulloblastomas and brain stem gliomas, in the pediatric population. Determining the appropriate array configurations to provide effective electric field coverage for the treatment of posterior fossa tumors is necessary for this expansion of applications [38].

Moreover, the application of TTFields to the treatment of extracranial tumors is also possible. Currently, Novocure, Ltd. is proceeding with the development of TTFields therapy for intrathoracic tumors, such as non-small cell lung cancer [39] and mesotheliomas [40], and intra-abdominal tumors, such as ovarian cancer [41], pancreatic cancer [42], and others [43]. Once the safety of TTFields therapy for the pediatric population is established, pediatric oncologists will have an overview of the expanded application of this therapy to various extra-cranial pediatric malignancies. However, preclinical studies are necessary to determine the appropriate intensity and frequency of the TTFields for each type of malignant tumor and to create an array specifically for use with children.

## 10. Conclusions

TTFields therapy is a novel, minimally toxic, and promising treatment modality for GBM and other malignancies throughout the body. Because of its unique anti-tumor mechanism, the development of multidisciplinary therapy in which TTFields therapy is combined with other treatment modalities should at least provide an additive benefit without the downside of increased toxicity. The development of pediatric neuro-oncological applications of this treatment relies on the availability of pediatric GBM cases, which as mentioned above, are very rare. Although the planned clinical trial of pediatric GBM will be very challenging to conduct, the authors hope that it will provide the key to innovating a variety of novel treatment strategies not only for pediatric GBM but also for all other forms of pediatric cancer.

## Figures and Tables

**Table 1:** Summary of efficacy results in pivotal trials of TTFields therapy.

Trial Code	Target Disease	Treatment Arm	N	Median PFS	*p*-Value	Median OS	*p*-Value	Ref.
EF-11	Recurrent GBM	TTF	120	9.3 weeks	0.24	6.6 months	0.27	[18]
		Chemotherapy	117	9.6 weeks		6.0 months		
EF-14	Newly diagnosed GBM	TTF + TMZ	210	7.1 months	0.0013	20.9 months	0.0042	[19]
		TMZ alone	105	3.9 months		16.0 months		

Abbreviations: GBM: glioblastoma multiforme, OS: overall survival, PFS: progression-free survival, TMZ: temozolomide, TTF or TTFields: tumor-treating fields.

**Table 2:** Pediatric case description of TTFields therapy.

	Age/Sex	Diagnosis	Disease Status	Concurrent Therapy	TTF Duration	Time to Progression	Survival After TTF	Toxicities	Ref.
1	13/F	GBM	1st recurrence	BV	13 mo	7 mo	-	Skin, grade 2	[32]
2	20/M	Anaplastic ODG	2nd recurrence	BV	1 mo	1 mo	11 mo	None	[33]
3	18/M	Epithelioid GBM	2nd recurrence	BV, CCNU	4 mo	3 mo	8 mo	None	
4	10/M	Gliomatosis cerebri	Newly diagnosed	BV	6 mo	6 mo	7 mo	None	
5	15/M	GBM	1st recurrence	BV, CCNU	6 mo	6 mo	10 mo	None	
6	11/M	DMG	Newly diagnosed	None	5 mo	5 mo	-	Skin, grade 2	
7	15/F	GBM	Newly diagnosed	TMZ	4 mo	4 mo	6 mo	None	[34]
8	9/M	GBM	Newly diagnosed	TMZ, everolimus	1 mo	1 mo	2 mo	None	
9	4/M	GBM	1st recurrence	BV, IRI, vorinostat	2 mo	2 mo	2 mo	None	
10	16/M	Gliomatosis cerebri	2nd recurrence	TMZ	3 mo	3 mo	6 mo	None	

Abbreviations: BV: bevacizumab, CCNU: lomustine, GBM: glioblastoma multiforme, IRI: irinotecan, mo: month(s), TMZ: temozolomide, ODG: oligodendroglioma.
